# Umbilical cord-derived mesenchymal stromal cells immunomodulate and restore actin dynamics and phagocytosis of LPS-activated microglia via PI3K/Akt/Rho GTPase pathway

**DOI:** 10.1038/s41420-021-00436-w

**Published:** 2021-03-15

**Authors:** Takeo Mukai, Elena Di Martino, Shunichiro Tsuji, Klas Blomgren, Tokiko Nagamura-Inoue, Ulrika Ådén

**Affiliations:** 1 Department of Women’s and Children’s Health, Karolinska Institutet, Stockholm, Sweden; 2 Department of Cell Processing and Transfusion, The Institute of Medical Science, The University of Tokyo, Bunkyo City, Japan; 3 Pediatric Oncology, Karolinska University Hospital, Stockholm, Sweden; 4 Department of Neonatology, Karolinska University Hospital, Stockholm, Sweden

**Keywords:** Mesenchymal stem cells, Cellular neuroscience

## Abstract

Microglia are the immune cells in the central nervous system surveying environment and reacting to various injuries. Activated microglia may cause impaired synaptic plasticity, therefore modulating and restoring them to neutral phenotype is crucial to counteract a pro-inflammatory, neurotoxic state. In this study, we focused on elucidating whether human umbilical cord (UC) -derived mesenchymal stromal cells (MSCs) can exert immunomodulatory effect and change the phenotype of activated microglia. Primary culture of microglia was activated by lipopolysaccharide (LPS) and was co-cultured with three lots of MSCs. We investigated immunomodulation, actin dynamics and phagocytic capacity of activated microglia, and examined change of Rho GTPase in microglia as the mechanism. MSCs suppressed the expression of IL-1β and pNFκB in LPS-activated microglia, and conversely elevated the expression of IL-1β in resting-surveying microglia with lot-to-lot variation. Morphological and phagocytotic analyses revealed that LPS stimulation significantly increased active Rho GTPase, Rac1, and Cdc42 levels in the microglia, and their morphology changed to amoeboid in which F-actin spread with ruffle formation. The F-actin spreading persisted after removal of LPS stimulation and reduced phagocytosis. On the other hand, MSC co-culture induced bimodal increase in active Rac1 and Cdc42 levels in LPS-activated microglia. Moreover, extended ruffles of F-actin shrinked and concentrated to form an actin ring, thereby restoring phagocytosis. We confirmed inhibition of the PI3K/Akt pathway attenuated F-actin dynamics and phagocytosis restored by MSCs. Overall, we demonstrated that MSCs immunomodulated microglia with lot-to-lot variation, and changed the phenotype of LPS-activated microglia restoring actin dynamics and phagocytosis by increase of active Rho GTPase.

## Introduction

Microglia, the immune cells of the central nervous system, are the first responders to brain injury, and are therefore critically involved in various injuries and diseases as key mediators^1^. Microglia are activated in response to brain injury, and are polarized toward inflammatory phenotype that enhances generation of pro-inflammatory mediators such as interleukin (IL)-1β and tumor necrosis factor (TNF)-α; however, they can also be polarized to the anti-inflammatory phenotype via mediators such as arginase 1 (Arg1) and transforming growth factor-β (TGF-β) during the resolution phase of brain inflammation^2,3^. Therefore, modulation of the phenotype of the microglia can be a novel therapeutic strategy for the treatment of neurological disorders accompanied by inflammation.

Recently mesenchymal stromal cells (MSCs) have attracted attention for their therapeutic potential in the treatment of neurological disorders. Indeed, MSCs have been used in several clinical studies for treating brain injuries^4,5^. MSCs have been isolated from several sources, including bone marrow, umbilical cord blood, adipose tissue, placenta, and umbilical cord (UC)^6–10^, and have been reported to perform immunoregulatory roles in inflammatory diseases^11^.

Kim et al. demonstrated that UC blood-derived MSCs mainly block the activation of microglia, which involved STAT1-mediated cytokine release^12^. However, whether UC-MSCs can immunomodulate and change the actin dynamics and phagocytic ability of activated microglia is not clear.

In this study, we aimed to elucidate whether human UC-MSCs can exert immunomodulatory effects and change the phenotype of activated microglia using three lots of human UC-MSCs.

## Results

### Characteristics of the primary culture of microglia and three lots of MSCs

**Fig. 1:**
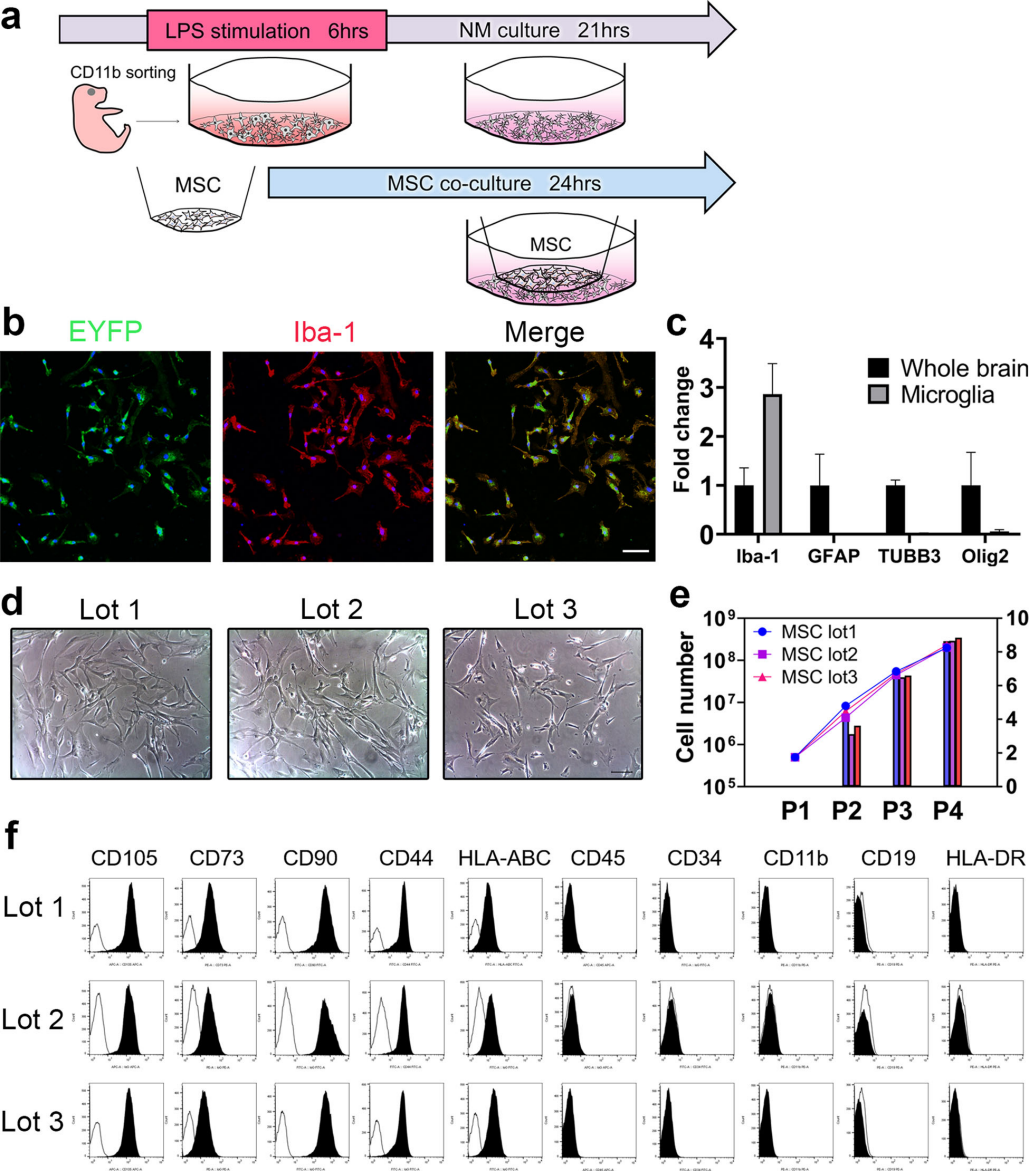
Experimental design and characteristics of primary culture of microglia and 3 lots of MSC. In this experiment, microglia were stimulated with LPS for 6 h and backed to culture in NM. In LPS + MSC group, co-cultured with MSC was started 3 h after LPS stimulation. The same procedure was performed without LPS stimulation for NM and NM + MSC groups (**a**). Immunocytochemistry of EYFP stained with anti-GFP antibody (green), Iba-1 (red) and Hoechst 33342 (blue) (scale bar = 50 μm) (**b**). The expression of Iba-1, GFAP, TUBB3 and Olig2 in microglia normalized to whole brain tissue by qPCR (**c**). Phase contrast images of 3 lots MSC (scale bar = 100 μm) (**d**). Cell number (line) and PDL (bar) of 3 lots MSC (**e**). Surface markers of MSC that positive for CD73, CD105, CD90, HLA-ABC, and CD44, and negative for CD45, CD34, CD11b, CD 19 and HLA–DR (**f**). Abbreviation: NM: normal medium, PDL: population doubling level.

The primary culture of microglia expressing EYFP under the *Cx3cr1* promoter expressed Iba-1 (Fig. 1b), and the proportion of EYFP-Iba-1 double positive cells was 99.3% (data not shown). The purity of the culture was also confirmed using qPCR, in which microglia expressed Iba-1, but not GFAP, TUBB3, or Olig2, compared to that in whole brain tissue (Fig. 1c).

All three lots of MSCs were spindle-shaped plastic-adherent cells, and PDL was similar between the three lots (Fig. 1d, e). Analysis of the surface markers of MSCs showed that they were all positive for CD73, CD105, CD90, HLA-ABC, and CD44, and negative for CD45, CD34, CD11b, CD 19, and HLA-DR as defined by the International Society for Cell Therapy^13,14^ (Fig. 1f).

### MSCs exerted immunomodulatory effect on LPS-activated microglia

**Fig. 2:**
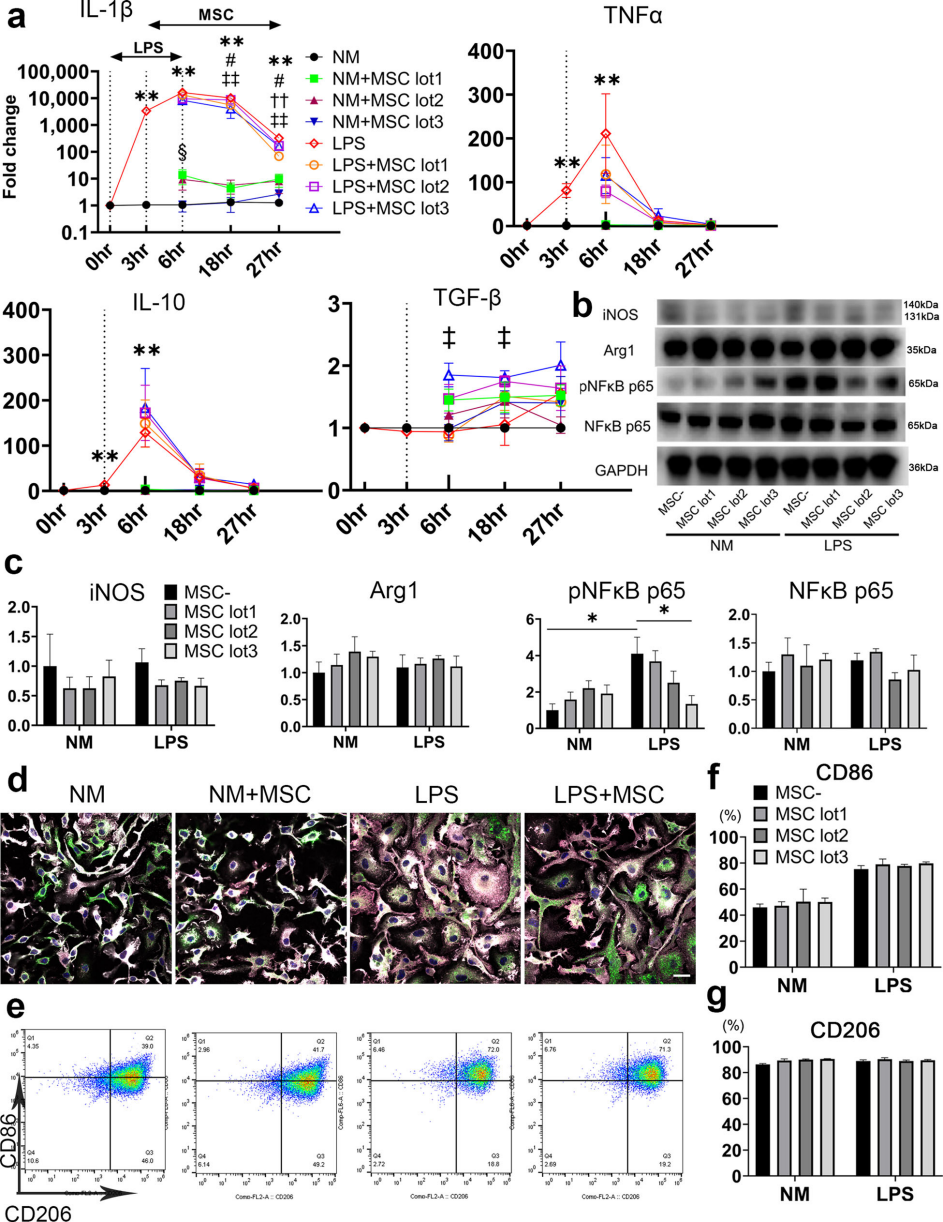
MSC exert immunomodulatory effect on LPS activated microglia. qPCR analysis of IL-1β, TNF-α, IL-10 and TGF-β genes in microglia. The expression of each gene was calculated by normalization to GAPDH. Data are presented as mean ± SEM. **p < 0.01; LPS group compared to NM group. ^§^*p* < 0.05; NM + MSC lot1 group compared to the NM group. ^#^*p* < 0.05; LPS + MSC lot1 group compared to the LPS group. ^††^*p* < 0.01; LPS + MSC lot2 group compared to the LPS group. ^‡‡^*p* < 0.01, ^‡^*p* < 0.05; LPS + MSC lot3 group compared to the LPS group (unpaired t-test and One-way ANOVA followed by Sidak’s multiple comparisons test for IL-10, and Mann–Whitney *U*-test and Dunn’s multiple comparisons test for IL-1β, TNF-α and TGF-β were used) (**a**). Western blotting results of NFκB, pNFκB, iNOS and Arg1. GAPDH was used as an internal control (**b**). Quantification of Western blotting. Data are presented as mean ± SEM with *p < 0.05 (One-way ANOVA followed by Sidak’s multiple comparisons test for iNOS and Dunn’s multiple comparisons test for Arg1, pNFκB and NFκB were used) (**c**). Immunocytochemistry of microglia stained with Iba-1 (white), CD86 (red), CD206 (green), and Hoechst 33342 (blue) (scale bar = 20 μm) (**d**). FACS analysis of microglia using CD86 and CD206 markers (**e**). Quantification of CD86 positive cells (**f**), and CD206 positive cells (**g**). Data are presented as mean ± SEM (One-way ANOVA followed by Sidak’s multiple comparisons test for CD206, and Dunn’s multiple comparisons test for CD86 were used).

To investigate the immunomodulatory effect of MSCs on microglia, we examined pro- and anti- inflammatory cytokine expression in microglia using qPCR (Fig. 2a). Compared to that in the NM group, IL-1β and TNF-α expression increased significantly 6 h after LPS stimulation and decreased after removal of LPS. Only the LPS + MSC lot 3 group showed the tendency to suppress the inflammatory peak (*p* = 0.08) of IL-1β. The expression of IL-1β decreased significantly and rapidly in LPS + MSC groups after 18 h in lot 1 and lot 3, and after 27 h in all the lots, whereas the expression of IL-1β in the NM + MSC lot 1 increased significantly compared to that in the NM group. On the other hand, TGF-β level did not increase after LPS stimulation, followed by a gradual decrease. Compared to that in the LPS group, only the LPS + MSC lot 3 group showed significant increase in the expression of TGF-β after 6 h and 18 h.

We also assessed the expression of pNFκB of the NFκB inflammatory pathway, and iNOS and Arg1 as pro-inflammatory and anti-inflammatory markers of microglia, respectively, after 6 h. Compared to that in the LPS group, the expression of pNFκB increased significantly after LPS stimulation, whereas it decreased significantly in the LPS + MSC lot 3 group. iNOS and Arg1 expression did not vary significantly between the LPS and LPS + MSC group (Fig. 2b, c).

To investigate the change in microglia polarization after LPS stimulation and co-culture with MSCs, we assessed the expression of surface markers, CD86 and CD206. Immunocytochemistry showed that compared to that in the NM group, the morphology of microglia apparently changed in the LPS group, whereas the LPS + MSC group showed thinner and more ramified morphology than the LPS group. More CD86-positive cells were observed in the LPS and LPS + MSC groups than in the NM and NM + MSC group (Fig. 2d). Results of flow cytometry showed that the number of CD86-positive cells tended to be higher in LPS group than in the NM group (*p* = 0.09), although the difference was not significant with and without MSC co-culture (Fig. 2e, f, g).

### Actin dynamics of LPS-activated microglia changed after co-culture with MSCs

**Fig. 3:**
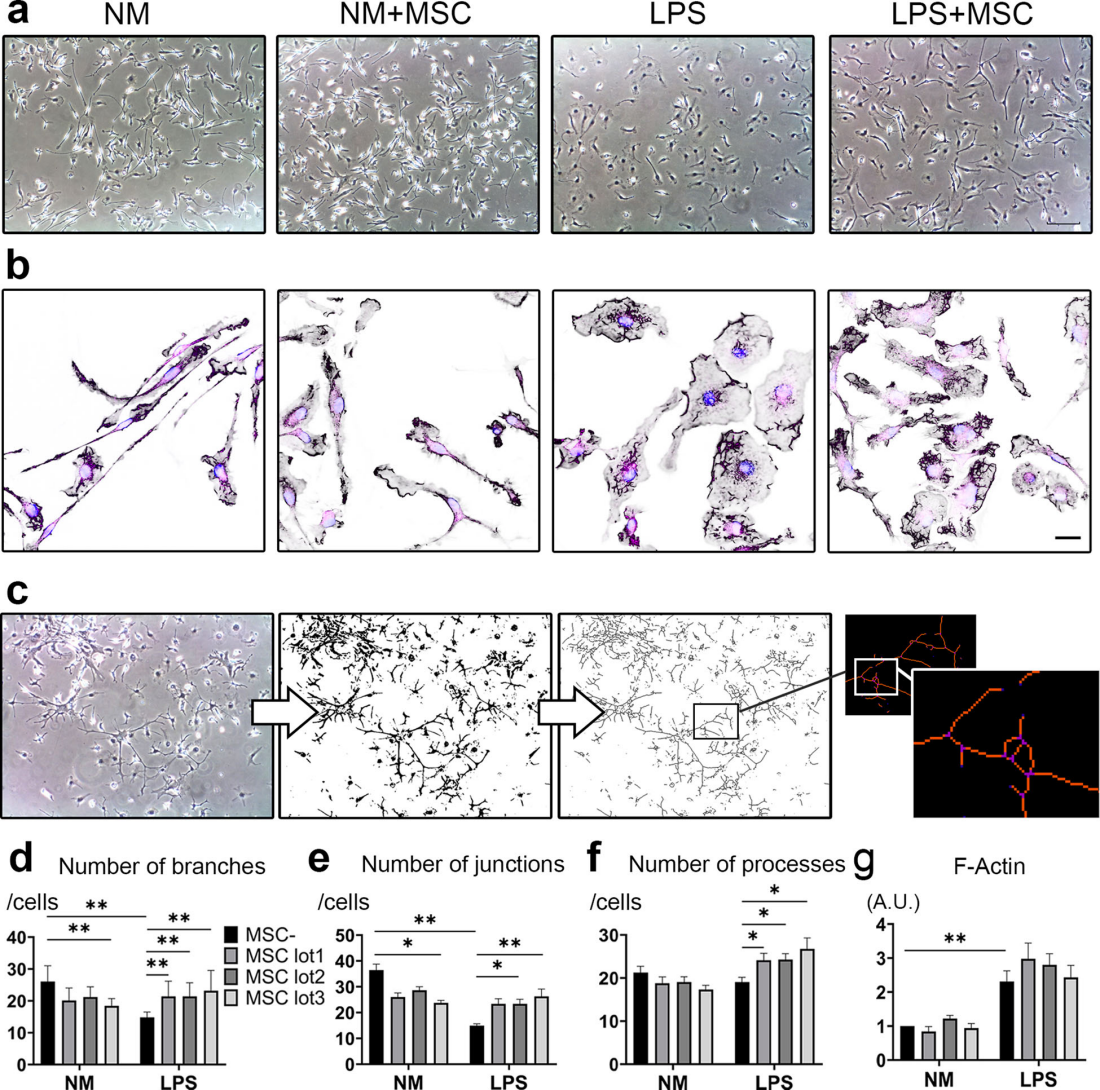
Actin dynamics of LPS activated microglia change after co-culture with MSC. Phase contrast images of four groups (scale bar = 100 μm) (**a**). Immunocytochemistry of F-Actin (black), vinculin (purple) and Hoechst 33342 (blue) (scale bar = 20 μm) (**b**). For skeleton analysis, the images underwent background subtraction, binarized and skeletonized for quantification (**c**). Quantification on the number of branches (**d**), junctions (**e**), processes (**f**), and F-Actin (**g**). (Dunn’s multiple comparisons test was used). Data are presented as mean ± SEM with **p < 0.01, *p < 0.05. Abbreviation: A.U., arbitrary units.

To investigate the morphological changes observed in the LPS + MSC group, we analyzed the actin dynamics of the microglia. Phase contrast images showed that microglia with thin and ramified morphology in the NM group became round and amoeboid in the LPS group, whereas they showed more elongated morphology and increased ramification in the LPS + MSC group (Fig. 3a).

Next, we investigated F-actin dynamics to assess the cause of the morphological change using immunocytochemistry for phalloidin and vinculin, the latter being a membrane-bound cytoskeletal proteins anchoring F-actin to the plasma membrane. In the NM group, linear F-actin filaments were stained with phalloidin along the narrow cell body; however, F-actin started to form ruffles along the membrane in the NM-MSC group. After LPS stimulation, F-actin spread with ruffle formation of the membrane, and F-actin concentration was observed in the lamellipodia. On the contrary, extended F-actin ruffles began to shrink and concentrate, resulting in morphological changes in the LPS + MSC group (Fig. 3b and Supplementary Fig. S1).

We quantified this morphological change caused by F-actin dynamics using skeletonization (Fig. 3c). Results showed that the number of branches and junctions were significantly lower in the LPS group than in the NM group. In addition, significant decrease in the number of branches and junctions was also observed in the NM + MSC groups with lot-to-lot variations. These reductions were recovered in the LPS + MSC groups (Fig. 3d, e). Furthermore, the number of processes was significantly higher in the LPS + MSC groups than in the LPS group (Fig. 3f). F-Actin quantification showed that LPS stimulation resulted in higher amounts of F-Actin compared to NM groups, but co-culture of MSC did not affect the total amount of F-actin (Fig. 3g).

### Co-culture with MSCs enhanced phagocytosis in LPS-activated microglia

**Fig. 4:**
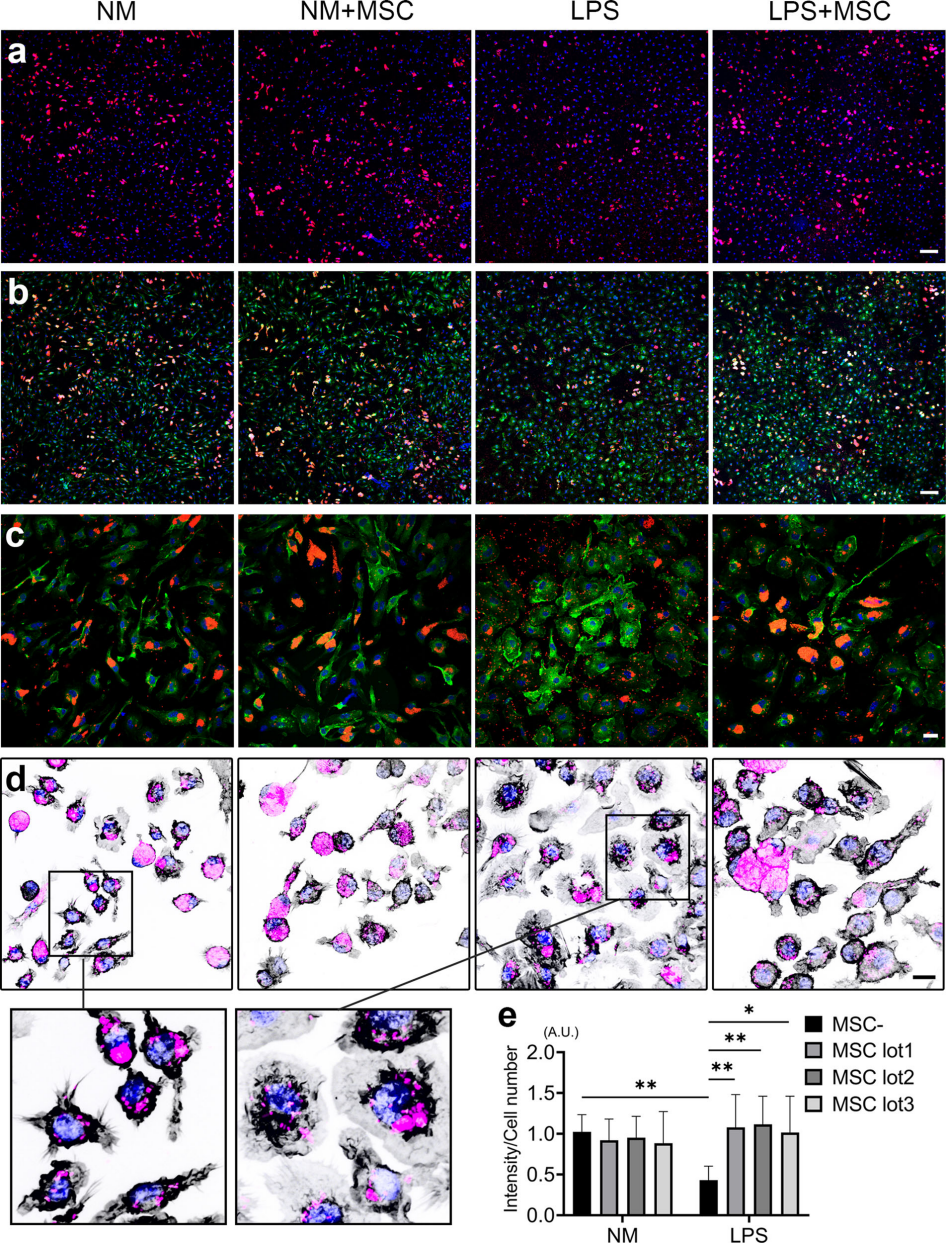
Co-culture with MSC enhance phagocytosis in LPS activated microglia. Immunocytochemistry of *E. coli* bioparticles (red) and Hoechst 33342 (blue) (**a**), and merge with EYFP (green) (**b**) (scale bar = 100 μm). High power field of Fig. 4b (**c**) (scale bar = 20 μm). F-Actin (black), *E. coli* bioparticles (red) and Hoechst 33342 (blue) (**d**) (scale bar = 20 μm). Quantification of phagocytosis (Dunn’s multiple comparisons test was used). Data are presented as mean ± SEM with **p < 0.01, *p < 0.05 (**e**). Abbreviation: A.U., arbitrary units.

We investigated how LPS stimulation and co-culture with MSCs affected phagocytosis. We found that LPS stimulation decreased phagocytosis of bioparticles, and that this reduction was recovered in the LPS + MSC group (Fig. 4a, b). We observed internalized particles in the NM groups and LPS + MSC group, whereas few clusters of particles were observed in the LPS group (Fig. 4c and Supplementary Fig. S2).

Furthermore, we investigated this phenomenon in terms of F-actin dynamics. In the NM, F-actin was well-organized at the edge of the cell membrane and formed a F-actin ring, and the bioparticles were along actin-dependent enhancement of the phagocytic cup or were internalized inside the ring. After LPS stimulation, F-actin was still near the center of the cell and was dispersed, which hampered ring formation in the LPS group. On the contrary, F-actin could form a F-actin ring, resulting in restored phagocytic capacity in the LPS + MSC group (Fig. 4d). Quantification showed that phagocytic capacity was significantly lower in the LPS group than in the NM group, whereas this attenuated capacity was significantly enhanced by co-culturing with MSCs in all lots (Fig. 4e).

### Co-culture with MSCs enhanced the activation and expression of Rho GTPase in LPS-activated microglia

**Fig. 5:**
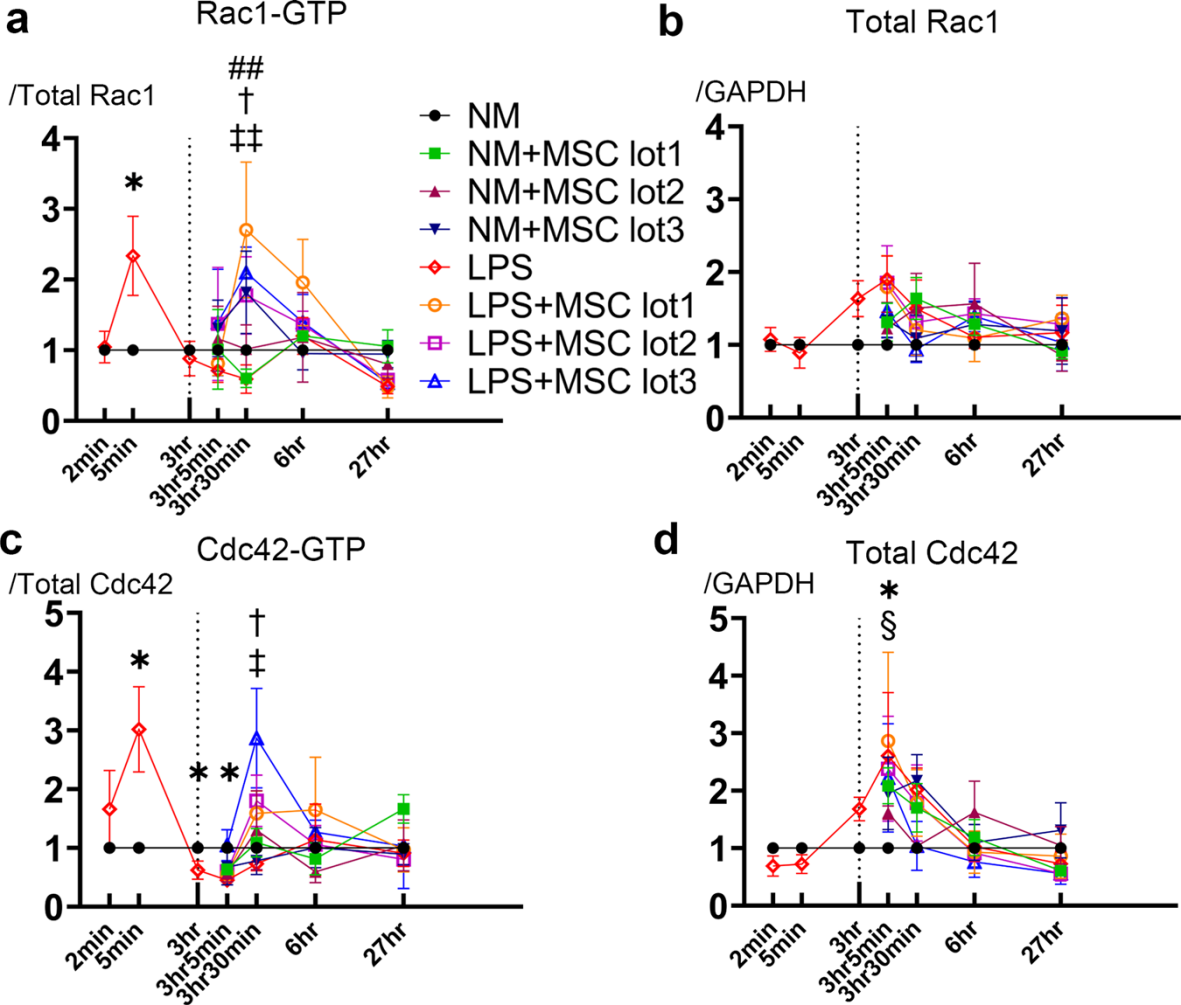
Co-culture with MSC enhance the activation and expression of Rho GTPase in LPS activated microglia. Western blotting images of Rho GTPase, Rac1 and Cdc42 in 2 min, 5 min, and 3 h after LPS stimulation and 5 min, 30 min, 3 h and 24 h after MSC co-culture. The data represent at least three individual experiments (**a**). Quantification of Western blotting showed by time course. *p < 0.05; LPS group compared to NM group. ^§^*p* < 0.05; NM + MSC lot1 group compared to the NM group. ^##^*p* < 0.01; LPS + MSC lot1 group compared to the LPS group. ^†^p < 0.05; LPS + MSC lot2 group compared to the LPS group. ^‡‡^*p* < 0.01, ^‡^*p* < 0.05; LPS + MSC lot3 group compared to the LPS group. The expression level of Rac1-GTP and Cdc42-GTP were calculated by normalization to total Rac1 and total Cdc42, and the expression level of total Rac1 and total Cdc42 were calculated by normalization to GAPDH. (Mann–Whitney U-test and Dunn’s multiple comparisons test were used) (**b**–**e**).

Microglial shape change and phagocytosis have been shown to be regulated by Rho GTPase^15–17^. Hence, we investigated the activation of Rho GTPase, Rac1, and Cdc42 (Fig. 5a). Compared to that in the NM group, the protein levels of both Rac1-GTP and Cdc42-GTP were significantly elevated 5 min after LPS stimulation, followed by decrease, especially for Cdc42-GTP. Subsequently, the expression levels of both Rac1-GTP and Cdc42-GTP were significantly higher in the LPS + MSC group 30 min after MSC co-culture than in the LPS group (Fig. 5b, d).

**Fig. 6:**
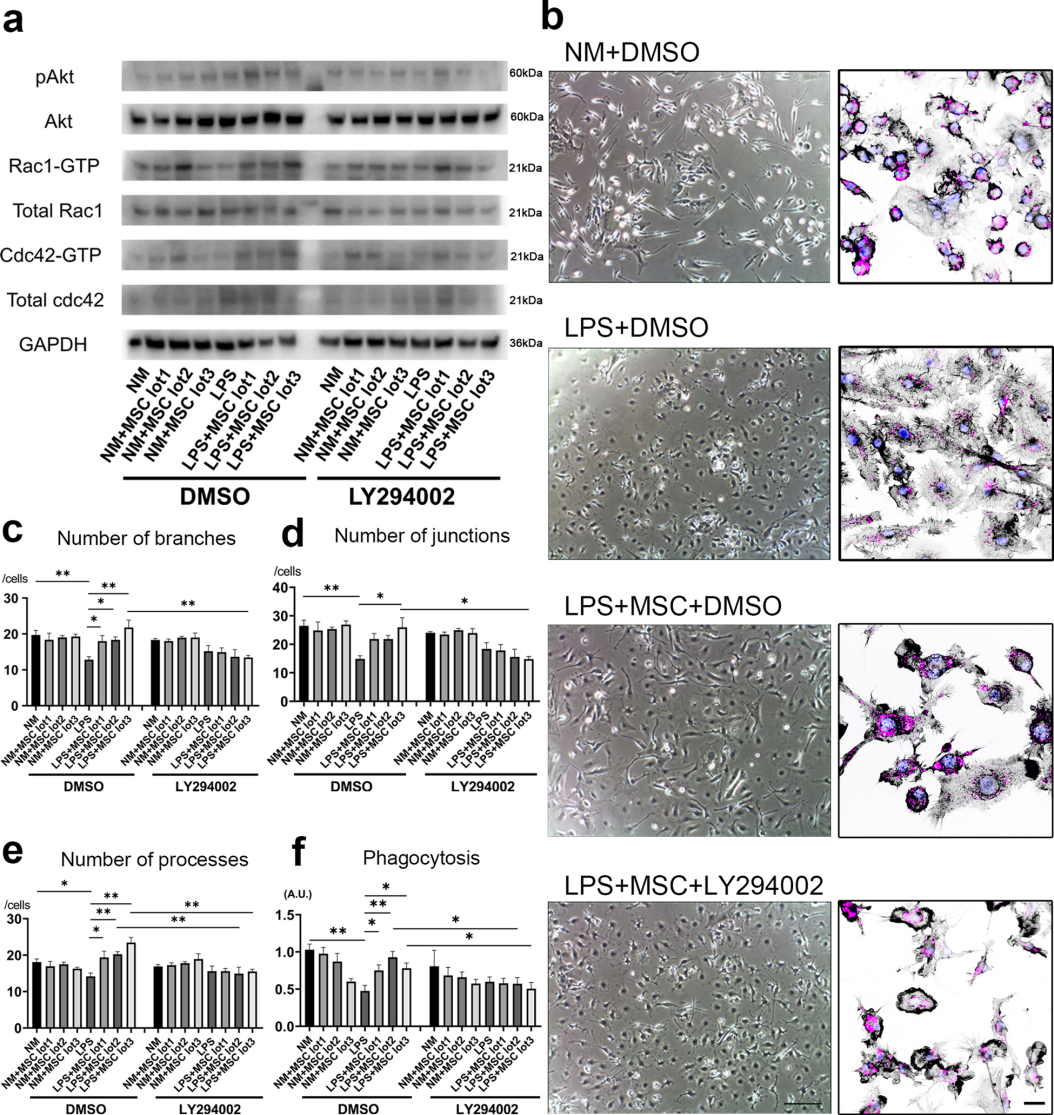
Inhibition of PI3K-Akt pathway blocked the activation of Rho GTPase and change of phenotype in LPS activated microglia. Western blotting images of pAkt, Akt, and Rho GTPase in 30 min after MSC co-culture. The data represent at least three individual experiments (**a**). Phase contrast images of four groups (scale bar = 100 μm) and immunocytochemistry of F-Actin (black), bioparticles (purple) and Hoechst 33342 (blue) (scale bar = 20 μm) (**b**). Quantification of the number of branches (**c**), junctions (**d**), processes (**e**), and phagocytosis (**f**). (Dunn’s multiple comparisons test were used). Data are presented as mean ± SEM with **p < 0.01, *p < 0.05. Abbreviation: A.U., arbitrary units.

In order to confirm whether the PI3K-Akt Rho GTPase pathway contributes to the changes in morphological and phagocytic properties, we used the PI3K-Akt inhibitor, LY294002, followed by morphological and phagocytic assays. At first, we confirmed LY294002 downregulated the expression of pAkt, Rac1-GTP, and Cdc42-GTP in the LPS + MSC group 30 min after MSC co-culture (Fig. 6a). In the morphological and phagocytic analyses, the LPS + MSC + LY294002 group showed lesser ramification and branch length than the LPS + MSC + DMSO group in phase contrast images. In addition, microglia in the LPS + MSC + LY294002 group showed distorted and disconnected F-actin ring formation, which resulted in lesser phagocytosis than in the LPS + MSC + DMSO group (Fig. 6b and Supplementary Fig. S3), demonstrating that restoration of phagocytosis by MSC co-culture was lost upon PI3K/Akt inhibition. Quantitative analyses showed that the number of branches, junctions, and processes decreased after LY294002 treatment (Fig. 6c, d, e). In addition, we confirmed that phagocytosis of microglia significantly decreased after PI3K/Akt inhibition in the LPS + MSC groups (Fig. 6f).

## Discussion

The present study demonstrated that MSCs can suppress inflammation in LPS-activated microglia, and conversely cause inflammation in NM-cultured microglia with lot-to-lot variation. Furthermore, this is the first study to characterize F-actin dynamics and phagocytosis of LPS-activated microglia using three lots of MSCs and a co-culture method. Consequently, LPS stimulation significantly increased active Rac1 and Cdc42 levels in the microglia, and their morphology changed to amoeboid, in which F-actin spread with ruffle formation. F-actin spreading persisted for 24 h after removal of LPS stimulation and resulted in reduction of phagocytosis. On the contrary, reduction in the levels of active Rac1 and Cdc42 were restored 30 min after MSC co-culture, and the extended F-actin ruffles began to concentrate and shrink, resulting in restored morphology and phagocytosis in the LPS + MSC group.

First, we established an in vitro model of LPS stimulation, followed by removal of LPS from the primary culture of microglia. Co-culturing with MSCs was initiated during LPS stimulation to analyze the feasibility of using MSCs as supportive cell therapy, in addition to radical treatment for neurological disorders accompanied by inflammation. We investigated paracrine effect of MSCs using co-culture system which does not have cell-to-cell contact in the present study. Several papers demonstrated that MSCs can inhibit the NFκB pathway by secreting anti-inflammatory cytokines such as TNF-α-stimulated gene/protein 6 (TSG-6), heme oxygenase (HO)-1, and prostaglandin E2^18–20^. Consistent with this observation, pNFκB was downregulated in the LPS + MSC group in our study. In contrast, we observed that some lots of MSCs may cause inflammatory response in resting microglia, indicating that MSCs should not be administered to healthy brain without inflammation.

Unlike previous reports that demonstrated bone marrow-derived MSCs can increase the expression of CD206 in the BV2 cell line^21^, UC-MSCs did not change the proportion of CD86- and CD206-positive microglia in our study, which may be due to the difference between primary culture and the BV2 cell line, and the source of MSCs used. Our study also showed that UC-MSCs were pro-inflammatory to resting microglia and anti-inflammatory to activated microglia, albeit with lot variance, and did not change this polarization significantly.

Recently, Rho GTPases, Rac1 and Cdc42, have been shown to regulate Fc receptor-mediated phagocytosis by controlling the different steps of membrane and actin dynamics, leading to particle engulfment^17,22^. Similar to that shown previously^23^, MSCs significantly changed the morphology of LPS-activated microglia in our study. Our results revealed that the number of branches, junctions, and processes decreased after LPS stimulation and significantly increased after MSC co-culture. This indicated that restoration of active Rac1 and Cdc42 by MSCs contributed to the branching process. Li et al. reported that enhanced Rac and Cdc42 activity selectively increased branch additions and retractions^24^, which supported our present results.

Next, we showed that the reduction in phagocytosis, which resulted from dispersed F-actin in the LPS group, was restored in the LPS + MSC group with formation of the actin ring. The observation that inhibition of the PI3K/Akt pathway, followed by downregulation of Rac1 and Cdc42, resulted in distortion of the F-actin ring and reduced phagocytosis indicated that the PI3K/Akt pathway was the main pathway via which MSCs activated the Rho GTPase. We have previously reported that intravenously administered MSCs constitutively secrete neurotrophic factors such as brain-derived neurotrophic factor (BDNF) and hepatocyte growth factor (HGF), both of which can activate the PI3K/Akt pathway^25^. Therefore, the activation of the PI3K/Akt pathway and restoration of Rac1 and Cdc42 by MSC co-culture in this study is consistent with our previous results. On the other hand, some reports showed that the Rho GTPase can be activated by signals from Toll-like receptor 4 (TLR4) via pathways acting upstream of Akt, such as high mobility group box-1 (HMGB1) or Janus kinase 2 (JAK2) signaling^26–28^. Our observation that the PI3K/Akt pathway was not activated only after LPS stimulation is consistent with the results of these previous reports considering that LPS binds to TLR4^29^. Our study also showed significant increase in total Cdc42 levels after LPS stimulation, which is in agreement with the results of a previous report showing that LPS increased total Cdc42 levels in polymorphonuclear leukocyte rafts^30^. Unlike previous report　that showed total Cdc42 level did not increase with MSC stimulation^15^, MSC co-culture increased total Cdc42 level in this study. It is presumed the different method of stimulation between co-culture and conditioned medium, and also different source of MSC are possibly responsible for this discrepancy.

However, some limitation should be noted. First, we tested three lots of MSCs derived from three different donors, but three lots are not a sufficient number to determine lot-to-lot or donor variation or conclusively demonstrate a correlation, although all the lots could restore actin dynamics and phagocytosis on LPS activated microglia by enhancement of Rho GTPase. Therefore, we should develop experiments in the present study using more lots of UC-MSCs from other donors and select some lots of UC-MSCs that exerts strong immune-modulation against activated microglia to use them as therapeutic potentials in the future. Second, the difference in chemokines and secretome secreted from MSC which influence microglia should be considered to elucidate phenotype of individual MSC lots. Because few studies have focused on the inflammation caused by MSC, the mechanism of inflammation by MSC requires further investigation. In addition, the effect of xenogeneic transplantation itself should be considered when it comes to cell-to-cell interaction.

In conclusion, this study showed that MSCs exerted their immunomodulatory effects and restored actin dynamics and phagocytosis of LPS-activated microglia via the PI3K/Akt/Rho GTPase pathway. Activated microglia may impair synaptic plasticity and cause neuronal apoptosis^31,32^; hence modulation and restoration of microglia to the resting phenotype are crucial for therapeutic purposes. Future studies should focus on the immunomodulatory signaling of MSCs toward microglia and the optimal criteria required for the selection of MSC lots for neurorestorative cell therapy for brain injuries.

## Materials and methods

### Microglia primary cultures

This study was approved by the ethical committee of the Karolinska Institutet, Stockholms norra djurförsöksetiska nämnd (approval number; N94/15 and N126/16) and performed in accordance with relevant guidelines and regulations (Swedish Animal Welfare Act 1988:543). The ARRIVE guidelines were followed for animal experiments.

We obtained Cx3cr1^CreER-EYFP+^ mice in which Cx3cr1 was replaced with yellow fluorescent protein (EYFP) as previously reported^33^. All mice were housed in a humidity-controlled room with a 12 h light-dark cycle with free access to food and water. Microglia were isolated from postnatal 2 (P2) to P3 newborn mice. No randomization or blinding was used for following experiments. Tissues were minced into pieces and incubated with enzymatic solution containing 0.01% papain (Sigma–Aldrich), 0.1% collagenase (Sigma–Aldrich), and 0.5% DNAse I (Sigma–Aldrich) in phosphate-buffered saline (PBS) at 37 °C for 20 min. Then, the enzymatic reaction was stopped with Dulbecco’s modified Eagle’s medium (DMEM)/F12 (Gibco), supplemented with 10% fetal bovine serum (FBS, Gibco). The cells were incubated with microglia growth medium consisting of DMEM/F12 supplemented with 10% FBS, 1% penicillin/streptomycin (Gibco), and recombinant murine macrophage colony stimulating factor (M-CSF) (PeproTech) for 10–14 days at 37 °C in the presence of 5% CO_2_; the medium was replaced twice a week. To separate microglia from the mixed glial cells, the cells were incubated with anti-mouse CD11b MACS antibody (Miltenyi Biotec) for 15 min and sorted via MACS sorting. The sorted microglia were seeded on poly-l-lysine-coated plate at the density of 1 × 10^5^ cells/ml.

### UC-MSCs preparation

Three lots of MSC isolation were performed in accordance with the recommendations of the Ethics Committee of the Institute of Medical Science, the University of Tokyo, Yamaguchi Hospital, and the NTT Medical Center Hospital, after obtaining written informed consent from all subjects in accordance with the Declaration of Helsinki. MSCs were isolated from three individual donors using previously reported methods^34^. Briefly, the UCs were collected after informed consent was obtained from pregnant women planning to undergo cesarean sections. Frozen-thawed UC tissues were minced into fragments for the improved explant culture method^35^. The tissue fragments were cultured with α-minimal essential medium (αMEM; Wako) supplemented with 10% FBS and 1% penicillin/streptomycin at 37 °C in the presence of 5% CO_2_. Fibroblast-like adherent cells that migrated from the UC tissue fragments were harvested using TrypLE Select and were defined as passage 1. These passage 1 MSCs were cryopreserved and transported by air from the Institute of Medical Science, the University of Tokyo, to Karolinska Institutet. The cells were passaged thrice at the Karolinska Institutet, after which, passage 4 MSCs were used for subsequent experimental analyses.

### Lipopolysaccharides (LPS) stimulation of microglia and co-culture with MSCs

Figure 1a shows the experimental design. For LPS stimulation, microglia cultured in microglia growth medium (normal medium; NM) at the density of 1 × 10^5^ cells/ml were stimulated for 6 h in NM supplemented with 5 ng/ml LPS from *E. coli* (Sigma–Aldrich). After 6 h of stimulation with LPS, the microglia were put back in culture in NM (LPS group). In the MSC co-culture group, co-culture with MSC using a 6-well Millicell cell culture insert (Merck Millipore) equipped with a 0.4-μm filter membrane was started 3 h after LPS stimulation. Microglia were cultured in the bottom chamber, while MSC suspended in NM were plated in the upper chamber at the density of 5 × 10^5^ cells/well and incubated overnight for 24 h at 37 °C in the presence of 5% CO_2_ (LPS + MSC group). The same procedure was performed without LPS stimulation for NM and NM + MSC groups. All cultures were maintained at 37 °C in a humidified atmosphere of 5% CO_2_.

LY 294002 was used as a PI3K-Akt inhibitor according to previously reported methods for inhibition experiments^15^. Briefly, microglia were incubated in NM supplemented with LY 294002 (Abcam) at a final concentration of 10 mM in 100% dimethyl sulfoxide (DMSO).

### Morphological analysis of microglia

Morphological analysis was performed 24 h after co-culture with MSCs as described previously^36,37^. For skeletal analysis, background subtraction was performed for the images, followed by binarization using the ImageJ version 1.49 software. Then, the images were skeletonized and analyzed for the quantification of the number of branches, junctions (if they have more than two neighbors), and processes (if they have less than two neighbors) using the “AnalyzeSkeleton” plugin. Finally, the values were normalized by the number of cells. For the analysis of filamentous actin (F-actin), the fluorescence intensity of F-actin in images on split channels were measured in ImageJ and normalized by the number of cells^38^. We performed morphological analysis from at least three randomly selected images per group in three independent experiments (containing total number of cells ranging from 1,089 to 1,521 cells per group).

### Quantitative polymerase chain reaction (qPCR)

Total RNA was isolated from cultured cells using the RNeasy kit (Qiagen). qPCR was performed using the Step-One-Plus real-time PCR machine (Applied Biosystems) and the Power SYBR Green PCR master mix (Applied Biosystems). The set-up condition was as follows: initial denaturation at 95 °C for 5 min, denaturation at 95 °C for 5 s, annealing at 60 °C for 10 s, and elongation for 30 s. The primer sequences used were as follows: IL-1β; 5′-TGCCACCTTTTGACAGTGATG-3′ (forward) and 5′-TGATGTGCTGCTGCGAGATT-3′ (reverse); TGF-β; 5′- TGCGCTTGCAGAGATTAAAA-3′ (forward) and 5′- CGTCAAAAGACAGCCACTCA-3′ (reverse); IL-10; Mm_Il10_1_SG (QT00106169, Qiagen); TNF-α; Mm_Tnf_1_SG (QT00104006, Qiagen); glyceraldehyde 3-phosphate dehydrogenase (GAPDH); Mm_Gapdh_1_SG (QT01658692, Qiagen); class III β-tubulin (TUBB3); Mm_TUBB3_1_SG (QT00124733, Qiagen); glial fibrillary acidic protein (GFAP); Mm_Gfap_1_SG (QT00101143, Qiagen); oligodendrocyte transcription factor 2 (Olig2); Mm_Olig2_1_SG (QT01041089, Qiagen). The expression of these markers was then normalized to that of GAPDH using the *ΔΔ*CT method. PCR was performed in triplicate for each sample in at least three independent experiments.

### Immunocytochemistry

Cultured microglia were washed twice with PBS and fixed with 4% paraformaldehyde for 30 min at room temperature. Then, the samples were blocked in 5% skim milk plus 0.3% Tween 20 (Sigma-Aldrich), and incubated overnight with primary antibodies at 4 °C. The primary antibodies included rabbit anti-Iba-1 (1:1000, Wako), chicken anti-green fluorescent protein (GFP) (1:500, Abcam), rat anti-CD86 (1:200, eBioscience), goat anti-CD206 (1:200, R&D Systems), and rabbit anti-vinculin (1:500, Cell Signaling Technology). The secondary antibodies used were donkey anti-rabbit IgG (Alexa Fluor® 555) (1:1000; Abcam), donkey anti-chicken IgG H&L (Alexa Fluor® 488) (1:1000; Abcam), donkey anti-rat IgG (Alexa Fluor® 594) (1:1000; Abcam), donkey anti-goat IgG (Alexa Fluor® 488) (1:1000; Abcam), and donkey anti-goat IgG (CF™ 633) (1:1000; Sigma Aldrich). Nuclei were counterstained with Hoechst 33342 (1:1000, Thermo Fisher). For F-actin staining, the cells were washed twice with PBS and fixed with 4% paraformaldehyde for 30 min at room temperature, followed by blocking in 5% skim milk plus 0.3% Tween 20 and incubation in the phalloidin-iFluor 488 reagent (1:1000, Abcam) for 1 h at room temperature. After incubation, the cells were washed five times with PBS. Images were acquired using a confocal microscope (Axio-observer Z1; Carl Zeiss microscopy, Germany) and analyzed using the ZEN 2.3 software (blue edition; Zeiss).

### Phagocytosis assay

To investigate phagocytotic ability, microglia were incubated with 20 mg/ml *E. coli* BioParticles conjugated to Alexa Fluor 594 (Invitrogen) 24 h after co-culture with or without MSC for 1 h at 37 °C in the presence of 5% CO_2_ according to the manufacturer’s protocols. After 1 h of incubation, the microglia were washed twice with PBS and subjected to immunocytochemistry as described above. Images were acquired using a confocal microscope and the fluorescence intensities were measured using ImageJ as described previously^39^. Phagocytotic analysis was performed from three randomly selected images per group in three independent experiments (containing total number of cells ranging from 3142 to 4739 cells per group).

### Flow cytometry analysis

The MSCs were stained using the following mouse monoclonal antibodies: fluorescein isothiocyanate (FITC)-conjugated anti-human CD90, phycoerythrin (PE)-conjugated anti-human CD105, allophycocyanin (APC)-conjugated anti-human CD73, FITC-conjugated anti-human CD44 (BD Biosciences), PE-conjugated anti-HLA-ABC (BD Biosciences), FITC-conjugated anti-HLA-DR (BD Biosciences), FITC-conjugated anti-human CD34 (BD Biosciences), PE-conjugated anti-human CD 11b (BD Biosciences), PE-conjugated anti-human CD 19 (BD Biosciences), and APC-conjugated anti-CD45 (BD Biosciences). Propidium iodide was used to identify and exclude the dead cells. The stained cells were acquired with a FACS Canto II flow cytometer (BD Biosystems) and analyzed using the FlowJo software (Tomy Digital Biology, Co. Ltd.). As for microglia, EYFP-expressing microglia were stained using APC-conjugated anti-mouse CD86 (BioLegend), PE-conjugated anti-mouse CD206 (ThermoFisher), and the Live/Dead™ fixable near-IR dead cell stain kit (Invitrogen). The stained cells were acquired with a Gallios flow cytometer (Beckman Coulter) and analyzed using the FlowJo software (Tomy Digital Biology, Co. Ltd.). Three independent FACS experiments were performed.

### Western blotting

Proteins were extracted from the cells at 2 min, 5 min, 3 h, 3 h, and 5 min (5 min after the co-culture with MSC), 3 h and 30 min, 6 h, and 27 h after LPS stimulation. Protein concentrations of the samples were measured using the Pierce™ bicinchoninic acid (BCA) protein assay kit (Thermo Scientific). After sodium dodecyl sulfate-polyacrylamide gel electrophoresis (SDS-PAGE), the polyvinylidene fluoride (PVDF) membranes were blocked using 1% Blocker BSA blocking buffer (Thermo Scientific) and incubated overnight at 4 °C with the primary antibodies. The primary antibodies included rabbit anti-inducible nitric oxide synthase (iNOS) (1:200, Abcam), rabbit anti- Arg1 (1:1000, Santa Cruz Biotechnology), rabbit anti-phospho-NFκB p65 (pNFκB) (1:1000, Cell Signaling Technology), rabbit anti-NFκB p65 (1:1000, Cell Signaling Technology), rabbit anti-pAkt (Ser473) (1:1000, Cell Signaling Technology), and rabbit anti-Akt (1:1000, Cell Signaling Technology). This was followed by incubation with horseradish peroxidase-conjugated anti-rabbit IgG (1:10000, Cell Signaling Technology) for 1 h at room temperature. The PVDF membranes were imaged using an enhanced chemiluminescence system with Pierce enhanced chemiluminescence (ECL) western blotting substrate (Thermo Scientific). The expression of the proteins was normalized to that of the NM group using ImageJ version 1.49 and quantifications were performed for at least three independent experiments.

### Rho GTPase activation assays

Rac1and Cdc42 activities were measured using a Rac1/Cdc42 activation assay kit (Millipore) according to the manufacturer’s protocols. Briefly, microglia were lysed on ice for 30 min in lyses buffer containing protease inhibitor cocktail and incubated with PAK-PBD agarose beads for 1 h at 4 °C, followed by three washes with washing buffer. The agarose beads were suspended in Laemmli buffer, loaded on an SDS-polyacrylamide gel, and analyzed using western blotting. The expression of active Rac1 and Cdc42 bound with GTP (Rac1-GTP and Cdc42-GTP, respectively) were normalized to total Rac1 and Cdc42 as described previously^15^.

### Statistical analysis

The Shapiro-Wilk test was used to check the normality of the data, and the data were analyzed using the unpaired *t*-test and one-way analysis of variance (ANOVA), followed by Sidak’s multiple comparison test for normally distributed data and the Mann–Whitney *U*-test and Dunn’s multiple comparison test for non-normally distributed data. The GraphPad Prism 8 software was used for all statistical analyses. Data are presented as mean±SEM and *p* < 0.05 was considered statistically significant.

## Supplementary information

Supplementary Figure 1

Supplementary Figure 2

Supplementary Figure 3

Supplementary Figure legends

## Data Availability

The datasets used and/or analyzed during the current study are available from the corresponding author on reasonable request.
