# Supplementary figures and images for: Umbilical cord-derived mesenchymal stromal cells immunomodulate and restore actin dynamics and phagocytosis of LPS-activated microglia via PI3K/Akt/Rho GTPase pathway
Source: Cell Death Discov. 2021 Mar 15;7:46. doi: 10.1038/s41420-021-00436-w (PMC7961004; doi:10.1038/s41420-021-00436-w)

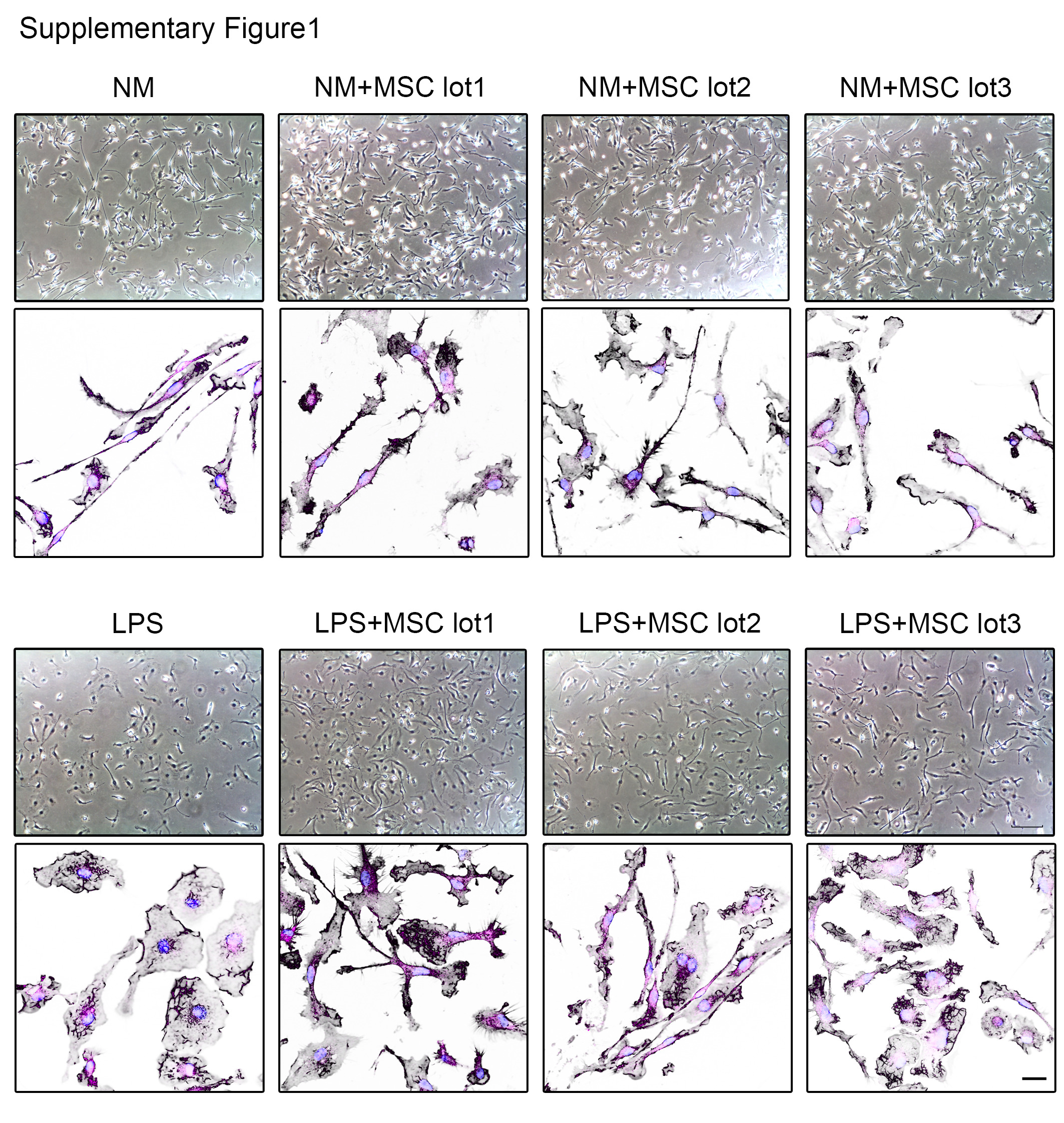

Supplement: Supplementary file 1 — Supplementary Figure 1 [file 41420_2021_436_MOESM1_ESM.tif]

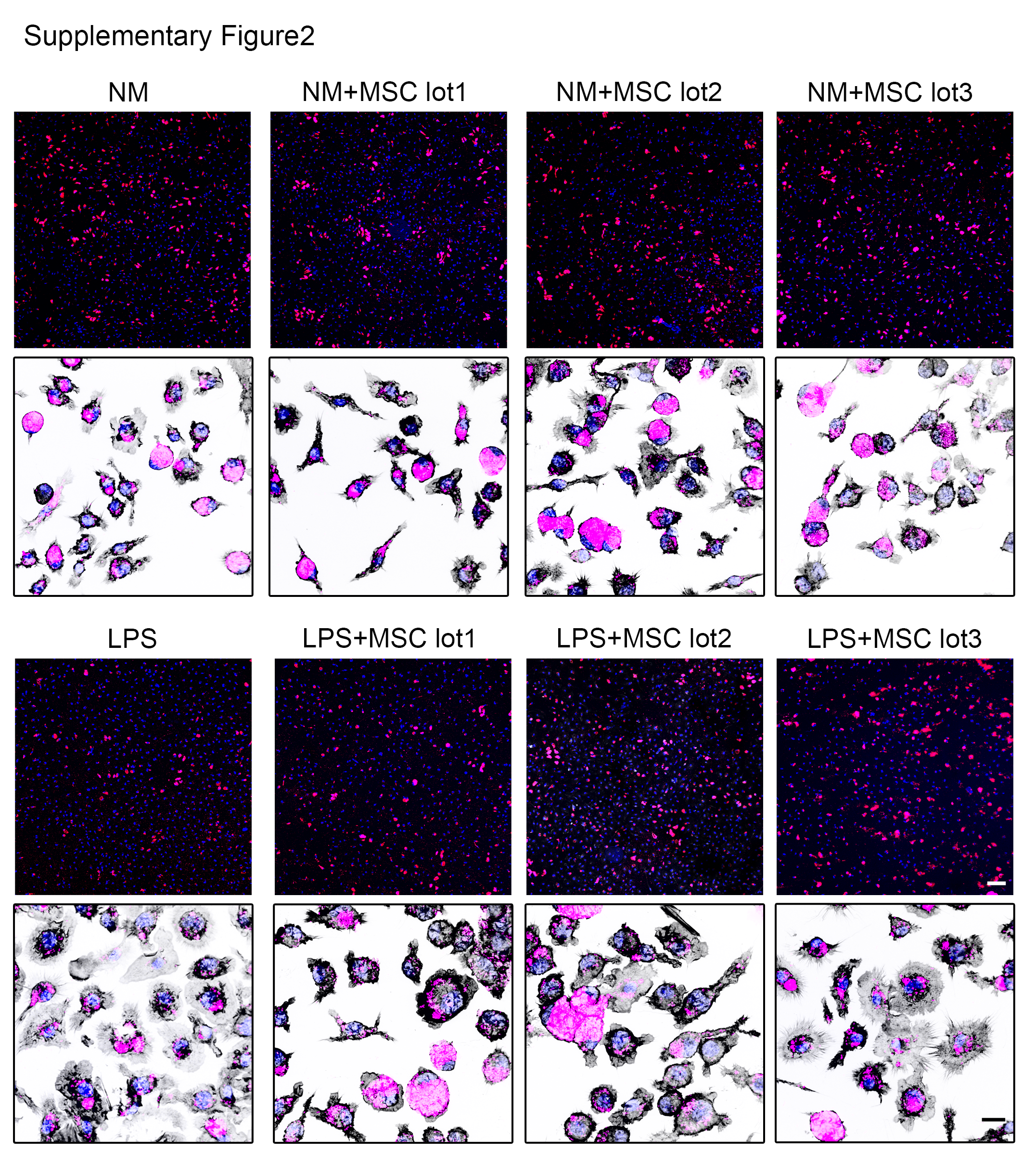

Supplement: Supplementary file 2 — Supplementary Figure 2 [file 41420_2021_436_MOESM2_ESM.tif]

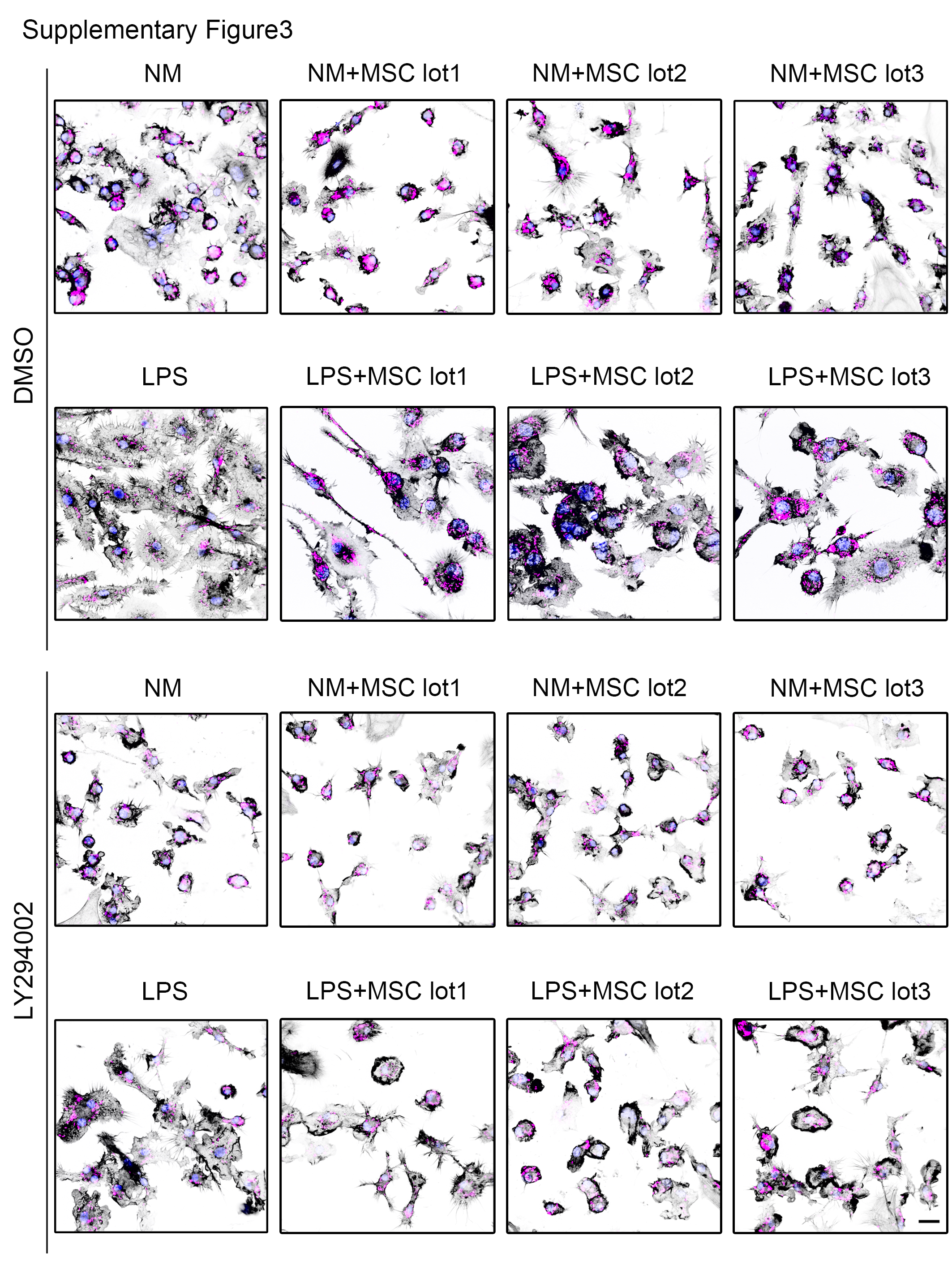

Supplement: Supplementary file 3 — Supplementary Figure 3 [file 41420_2021_436_MOESM3_ESM.tif]
