# Supplementary material for: Umbilical cord-derived mesenchymal stromal cells immunomodulate and restore actin dynamics and phagocytosis of LPS-activated microglia via PI3K/Akt/Rho GTPase pathway
Source: Cell Death Discov. 2021 Mar 15;7:46. doi: 10.1038/s41420-021-00436-w (PMC7961004; doi:10.1038/s41420-021-00436-w)
Supplement: Supplementary file 4 — Supplementary Figure legends [file 41420_2021_436_MOESM4_ESM.docx]

**Supplementary Figure Legends**

Supplementary Figure 1. Actin dynamics of LPS activated microglia change after co-culture with MSC

Phase contrast images of eight groups (scale bar = 100 μm), and immunocytochemistry of F-Actin (black), vinculin (purple) and Hoechst 33342 (blue) (scale bar = 20 μm)

Supplementary Figure 2. Co-culture with MSC enhance phagocytosis in LPS activated microglia

Immunocytochemistry of E. coli bioparticles (red) and Hoechst 33342 (blue) (upper row, scale bar = 100 μm), and F-Actin (black), E. coli bioparticles (red) and Hoechst 33342 (blue) in eight groups (lower row, scale bar = 20 μm).

Supplementary Figure 3. Inhibition of PI3K-Akt pathway blocked the change of phenotype in LPS activated microglia

Immunocytochemistry of F-Actin (black), bioparticles (purple) and Hoechst 33342 (blue) in eight groups cultured with DMSO or LY294002 (scale bar = 20 μm).
